# Wnt Antagonist Secreted Frizzled-Related Protein 4 Upregulates Adipogenic Differentiation in Human Adipose Tissue-Derived Mesenchymal Stem Cells

**DOI:** 10.1371/journal.pone.0118005

**Published:** 2015-02-25

**Authors:** Malini Visweswaran, Luca Schiefer, Frank Arfuso, Rodney J. Dilley, Philip Newsholme, Arun Dharmarajan

**Affiliations:** 1 School of Biomedical Sciences, Curtin Health Innovation Research Institute, Curtin University, Perth, Western Australia, Australia; 2 University of Lübeck, Lübeck, Germany; 3 Ear Sciences Centre, University of Western Australia and Ear Science Institute Australia, Perth, Western Australia, Australia; Yong Loo Lin School of Medicine, National University of Singapore, SINGAPORE

## Abstract

With more than 1.4 billion overweight or obese adults worldwide, obesity and progression of the metabolic syndrome are major health and economic challenges. To address mechanisms of obesity, adipose tissue-derived mesenchymal stem cells (ADSCs) are being studied to detail the molecular mechanisms involved in adipogenic differentiation. Activation of the Wnt signalling pathway has inhibited adipogenesis from precursor cells. In our study, we examined this anti-adipogenic effect in further detail stimulating Wnt with lithium chloride (LiCl) and 6-bromo indirubin 3’oxime (BIO). We also examined the effect of Wnt inhibition using secreted frizzled-related protein 4 (sFRP4), which we have previously shown to be pro-apoptotic, anti-angiogenic, and anti-tumorigenic. Wnt stimulation in LiCl and BIO-treated ADSCs resulted in a significant reduction (2.7-fold and 12-fold respectively) in lipid accumulation as measured by Oil red O staining while Wnt inhibition with sFRP4 induced a 1.5-fold increase in lipid accumulation. Furthermore, there was significant 1.2-fold increase in peroxisome proliferator-activated receptor gamma (PPARγ) and CCAAT/enhancer binding protein alpha (C/EBPα), and 1.3-fold increase in acetyl CoA carboxylase protein levels. In contrast, the expression of adipogenic proteins (PPARγ, C/EBPα, and acetyl CoA carboxylase) were decreased significantly with LiCl (by 1.6, 2.6, and 1.9-fold respectively) and BIO (by 7, 17, and 5.6-fold respectively) treatments. These investigations demonstrate interplay between Wnt antagonism and Wnt activation during adipogenesis and indicate pathways for therapeutic intervention to control this process.

## Introduction

Obese and overweight conditions are becoming progressively prevalent and are a major health challenge worldwide [1]. Apart from significantly affecting quality of life [2], obesity has several significant co-morbidities such as hypertension, type 2 (T2) diabetes, cardiovascular disease, and increased cancer risk [3,4]. Hence, understanding the molecular mechanisms contributing to the obese condition, such as increased proliferation of existing pre-adipocytes or increased differentiation from their precursor mesenchymal stem cells (MSCs), becomes significant in order to develop novel therapeutic controls for obesity. Adipose tissue-derived mesenchymal stem cells (ADSCs) are attractive candidates in studying mechanisms involved in adipose biology, taking into account their strong adipogenic differentiation capability when compared to MSCs derived from other sources such as bone marrow [5–8]. ADSCs also have osteogenic and chondrogenic differentiation capability, fulfilling their MSC characteristic [5,6].

While adipogenic differentiation has been shown to be regulated by different signalling pathways, the Wnt signalling pathway is considered a key player regulating adipogenesis [9–12]. This pathway is controlled at various phases by an array of Wnt activating and inhibiting molecules. The secreted frizzled-related proteins (sFRPs) are major Wnt antagonists that inhibit Wnt signalling by binding to either the Wnt ligand or the Frizzled receptor, or both [13,14]. Although the role of Wnt activators in determining the fate of adipocyte precursors in murine models has been demonstrated [9], there are very few reports about the role of the Wnt antagonists in determining mesenchymal stem cell (MSC) differentiation. An inhibitory effect on adipocyte lipid accumulation has been shown by Wnt activating molecules such as Wnt 10b, glycogen synthase kinase 3β inhibitors such as lithium chloride (LiCl) [9], and 6-bromo indirubin 3’oxime (BIO) [15].

So far there are no studies examining the impact of continuous supplementation of exogenous sFRP4 on adipogenic differentiation. Hence, in this study, we examined the effects of Wnt antagonism using recombinant secreted frizzled-related protein 4 (sFRP4) protein with regard to changes in cell morphology, lipid droplet accumulation, and adipogenesis-specific protein expression in ADSCs. Additionally, the inhibitory effect of the pharmacological Wnt activators, such as LiCl and BIO, on the levels of adipogenesis-specific proteins has been revealed.

## Materials and Methods

### Cell culture

Human adipose tissue-derived mesenchymal stem cells (ADSCs; Cat No: PT-5006) were purchased from Lonza Corporation, Australia. ADSCs were cultured in growth media (Low glucose DMEM (Invitrogen) media, 10% FBS (Serana), and 1% Penicillin/Streptomycin (Hyclone)) and were subcultured using TrypLE Express (Invitrogen) to subsequent passages. All the experiments were carried out between passages 3–6.

### Characterization of MSCs by adherence, surface markers, and tri-lineage differentiation

The plastic adherence property of MSCs was observed by culturing in appropriate media at 37°C in the presence of 5% CO_2_. The surface markers had been previously analysed by flow cytometric characterization (Lonza). Further, for characterising the multipotent property of ADSCs, tri-lineage differentiation was performed into adipogenic, osteogenic, and chondrogenic lineages. Briefly, the cells were seeded at the appropriate seeding densities, grown to 90% confluence in growth media, and then replaced by the respective differentiation media (Invitrogen) for specific durations. Undifferentiated ADSCs maintained in basal growth media served as control.

At the end of the differentiation period, lineage-specific staining was performed to visualise the differentiation and observed using bright field microscopy. Briefly, cells were fixed with 4% paraformaldehyde for 30 minutes, and rinsed with phosphate buffered saline (PBS). Following fixation, lineage-specific staining methods such as Oil Red O, alizarin red/von Kossa, and alcian blue were used for detecting adipogenic, osteogenic, and chondrogenic lineages respectively.

### Treatment doses for Wnt activators and Wnt antagonists

The following regulators of the Wnt signalling pathway were used: Wnt activators (i) LiCl, and (ii) BIO, and the Wnt antagonist sFRP4. The doses of these molecules required for the differentiation assay were standardised using a cell viability assay with methyl thiazolyl tetrazolium (MTT). The surface-adherent ADSCs were treated for 48hours with different doses of LiCl (ranging from 1mM-20mM), BIO (ranging from 0.5μM-10μM), and sFRP4 (ranging from 100pg/mL to 1μg/mL). Following the treatment, 10μL of MTT were added to the media for a further incubation of 4 hours at 37°C, and the resulting formazan crystals were dissolved in 100μL DMSO. The absorbance read at 555nm corresponded to cell viability.

### Adipogenic differentiation

For adipogenic differentiation, ADSCs in passage 4 were seeded at 10,000 cells/cm^2^, and allowed to grow to 90% confluence. On Day 5 post-seeding, the adipogenic induction was initiated by replacing basal growth media with adipogenic differentiation media (Stempro adipogenic differentiation kit, Invitrogen) in treatment wells, while control ADSCs was maintained in their growth media. The adipogenic media were individually supplemented with appropriate doses of the Wnt regulators LiCl, BIO, and sFRP4 (as standardised by the MTT cell viability assay) during the period of differentiation. Later, a combination treatment containing both BIO and sFRP4 was also performed to observe the interaction between the Wnt activator and antagonist together on adipogenesis. The media were replenished every 3–4 days. Morphological observations for accumulation of lipid droplets in all treatment conditions were recorded and photographed at regular intervals throughout the adipogenic differentiation period. On Day 7 post-induction, cells were analysed for lipid accumulation by Oil red O staining and were harvested to detect the protein expression of adipogenic markers. 24 well-plates were used for histochemical staining while 6 well-plates were used for protein extraction.

### Oil Red O staining and quantification

A working solution of Oil Red O stain was prepared by adding 30mL stock solution (0.5% Oil Red O in 100% isopropanol (Sigma)) with 20mL distilled water. On day 7, the cells were fixed with 4% paraformaldehyde for 30 minutes, rinsed with PBS, and stained with the Oil Red O working solution for 30 minutes. Cells were rinsed with PBS, observed for the stained intracellular lipid droplets, and photographed using bright field inverted Nikon microscope and Nikon software. Quantification of the stained area was performed by eluting the stain off the plate by incubation with 100% isopropanol for 1 hour, followed by measuring the absorbance of the elutes at 510nm using an EnSpire multimode plate reader (Perkin Elmer).

### Western Blot analysis

The whole-cell lysates of ADSCs at Day 7 of differentiation were obtained using a 1X RIPA lysis buffer and quantified using a BCA assay (Pierce) as per the manufacturer’s instructions. Briefly, 20μg of protein were loaded onto a 4–12% Bis-Tris SDS-PAGE gel (Invitrogen) and separated at 165V for 35 minutes. Following electrophoresis, the protein bands were electro-transferred onto the nitrocellulose membrane using iBlot transfer stacks and device (Invitrogen), followed by blocking the membrane for 1 hour using 5% non-fat dry milk. Incubation of the blot with anti-human rabbit primary antibodies specific for proteins regulating adipogenesis were carried out overnight at 4°C on a shaker. The antibodies used were: PPARγ, CCAAT enhancer binding protein alpha (C/EBPα), and acetyl CoA carboxylase (ACC) (Adipogenesis marker antibody sampler kit, Cat No: 12589, Cell Signaling). The blots were also probed with an antibody against β-actin (Cell Signaling) as the loading control. All primary antibodies were used at 1:1000 dilution in 5% BSA prepared in PBS containing 0.1% tween-20 (PBST). This was followed by anti-rabbit HRP-conjugated secondary antibody incubation at 1:2000 prepared in 5% milk prepared in PBST for 1 hour at room temperature and chemiluminescence detection (Western Lightning Plus ECL kit, Perkin Elmer). The washes following blocking, primary, and secondary antibody incubation steps were performed using PBST. Densitometry of the detected bands was used to quantify the protein expression with Image Lab (BioRad) software, and the resulting band intensities were normalised to β-actin.

### Data analysis

All experiments were performed in triplicates and the mean values ± SEM were calculated. A Student’s t-test was used to compare the values of the treatment conditions with the untreated adipogenic control. A p value of less 0.05 was considered statistically significant. The images showing cell morphology and staining has been grayscaled using Adobe Photoshop CS6 and adjusted for brightness and contrast using Microsoft Office Picture Manager.

## Results

### ADSC characterization and tri-lineage differentiation

**Fig 1:**
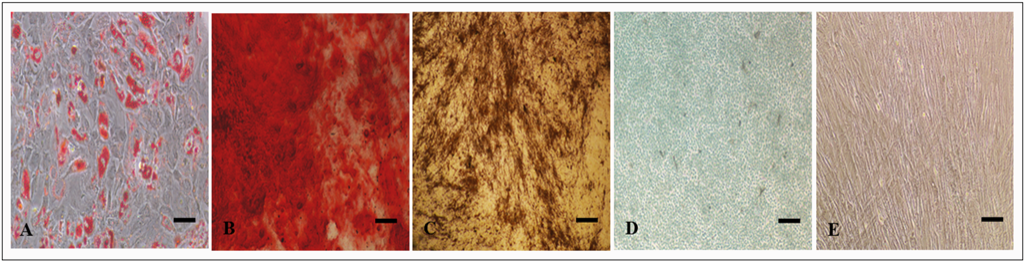
Differentiation of ADSCs visualised by staining techniques. (A) Intracellular lipid globules of adipogenically differentiated ADSCs stained positively by Oil Red O (B) Calcium deposition of osteogenically differentiated ADSCs stained positively by Alizarin Red, (C) Mineralization of osteogenically differentiated ADSCs stained positively by Von Kossa, (D) Glycosaminoglycan deposition of chondrogenically differentiated ADSCs stained positively by Alcian blue, and (E) Fibroblast morphology of undifferentiated ADSCs. Scale bar = 10μM.

To confirm the multi-potent nature of these ADSC cultures, their differentiation into adipogenic (7 days), osteogenic (28 days), and chondrogenic (21 days) lineages was examined. The results of the lineage-specific staining at the end of the indicated differentiation period showed the presence of lipid droplets, calcium mineralisation and proteoglycan deposition in the adipogenic, osteogenic, and chondrogenic differentiation respectively (Fig. 1) confirming the ADSC tri-lineage differentiation capacity.

### Standardization of treatment doses for the Wnt activators and Wnt antagonist

**Fig 2:**
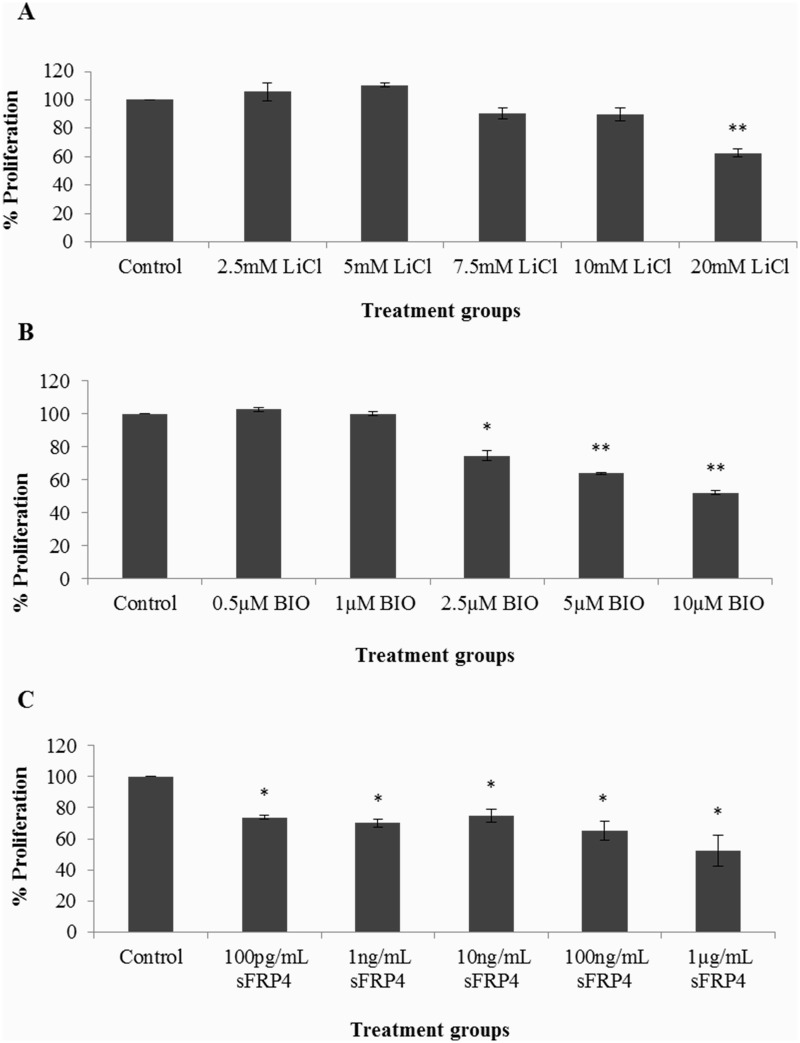
Dose response of ADSCs with Wnt regulators. In the presence of Wnt activators (A) LiCl, (B) BIO, and (C) Wnt antagonist sFRP4 (* p<0.05 and ** p<0.001).**iiiiiiivviv**

ADSCs were treated for 48 hours with various doses of the Wnt regulators—LiCl, BIO, and sFRP4, and the resulting cell viability analysed using an MTT assay. The proliferation of ADSCs was inhibited in a dose-dependent manner (Fig. 2A and 2B). As a result, the doses of these Wnt regulators selected for subsequent experiments were 10mM (LiCl), 0.5μM (BIO), 100pg/mL (sFRP4) and 1ng/mL (sFRP4), in accordance with the minimum lethality caused by the dose.

### Adipogenic differentiation of ADSCs in the presence of Wnt regulators

Adipogenic differentiation was induced on the ADSCs at 90% confluence and continued for 7 days. The adipogenic media were supplemented with the selected doses of LiCl, BIO, and sFRP4. The untreated adipogenic media served as control for the adipogenesis. Also, undifferentiated ADSCs cultured in the basal expansion media served as the non-induced control. The cells were frequently observed for morphology changes and lipid droplet formation.

**Fig 3:**
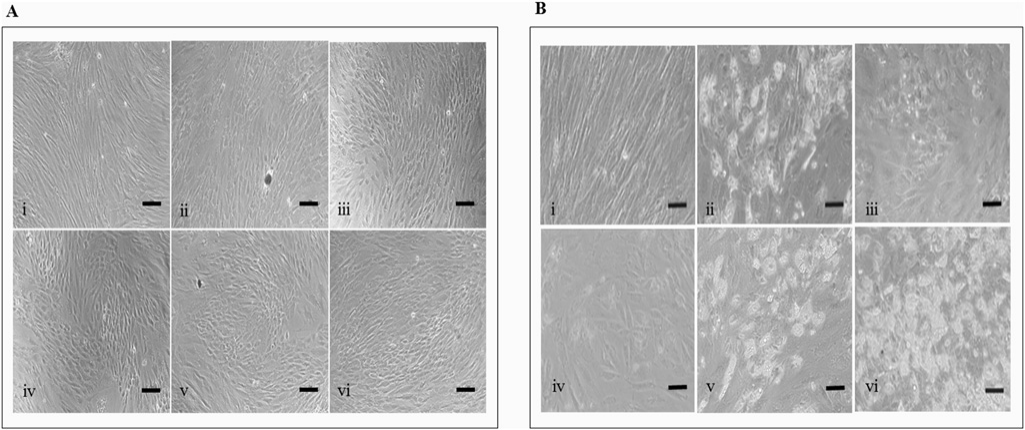
Morphology of ADSCs during adipogenic differentiation. At (A) Day 1 and (B) Day 7. Treatment conditions were (i) non-induced control media, (ii) adipogenic control, (iii) 10mM LiCl, (iv) 0.5μM BIO, (v) 100pg/mL sFRP4, and (vi) 1ng/mL sFRP4 in both (A) and (B). Scale bar = 10μM.

Irrespective of the treatment conditions, the transformation of morphology from a spindle-like shape into a rounded shape was initiated by Day 1 (Fig. 3A). In all the treatment conditions, the lipid droplets started appearing at Day 4, but by Day 7 there was a further increase seen in lipid droplet content of the sFRP4(1ng/mL)-treated compared to the adipogenic control groups (Fig. 3B). In the treatment groups containing the LiCl and BIO, adipogenesis remained low with lesser lipid droplet content (Fig. 3B). Non-induced ADSCs retained their fibroblastic morphology with no lipid accumulation.

### Oil Red O staining and quantification of the degree of adipogenesis

**Fig 4:**
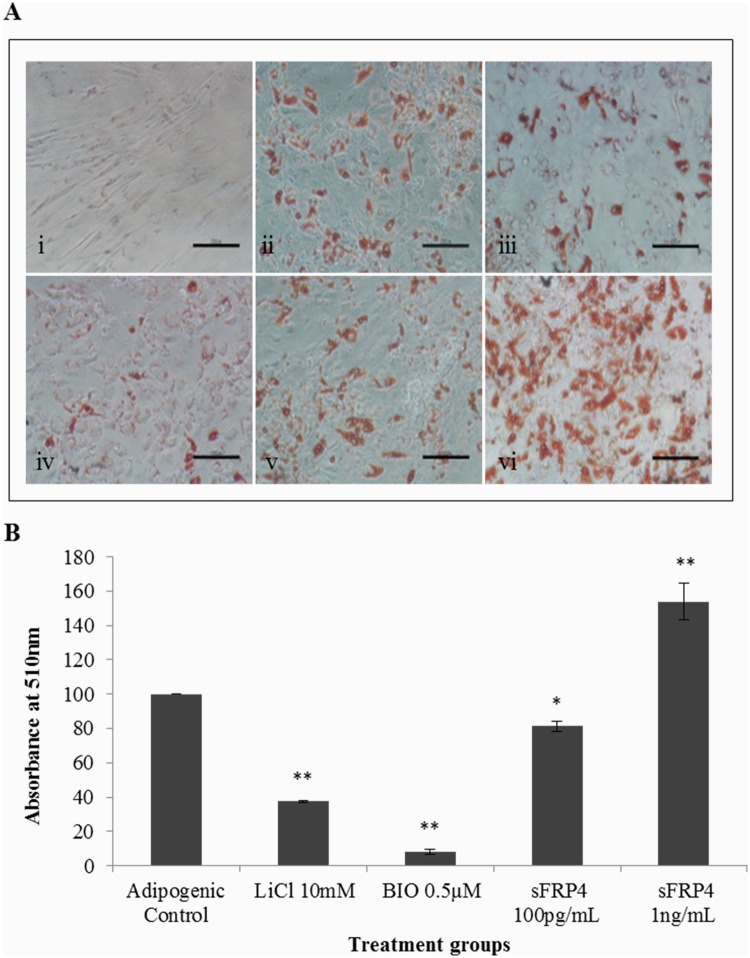
Oil Red O Staining and quantification on Day 7. (A) Microscopic observations of the stained lipid droplets. Treatment conditions were (i) non-induced control, (ii) adipogenic control, (iii) 10mM LiCl, (iv) 0.5μM BIO, (v) 100pg/mL sFRP4, and (vi) 1ng/mL sFRP4. Scale bar = 250μM (B) Quantification of the stained lipid droplets were performed using the eluted Oil red O stain via measuring absorbance at 510nm. The readings were normalised to background values of non-induced control ADSCs. The values of all treatment conditions were compared to the adipogenic control group (* p<0.05 and ** p<0.001).

Oil Red O staining was performed on Day 7 of adipogenic differentiation, and the stained (red) intracellular lipid droplets were visualised using bright field microscopy. The least lipid droplet accumulation was observed in the groups with LiCl, and BIO and the highest observed in sFRP4 1ng/mL group (Fig. 4A).

To further quantify the total stained area in each treatment condition, Oil red O was eluted from the stained wells using 100% isopropanol and absorbance measured at 510nm. While the Wnt activators LiCl and BIO caused a 2.7-fold and 12-fold decrease in the lipid droplet content, sFRP4 at 1ng/mL produced a 1.5-fold increase in lipid accumulation (Fig. 4B).**Aiviiiivviii**

### Effect of the Wnt regulators on adipogenic marker protein expression of differentiated ADSCs

**Fig 5:**
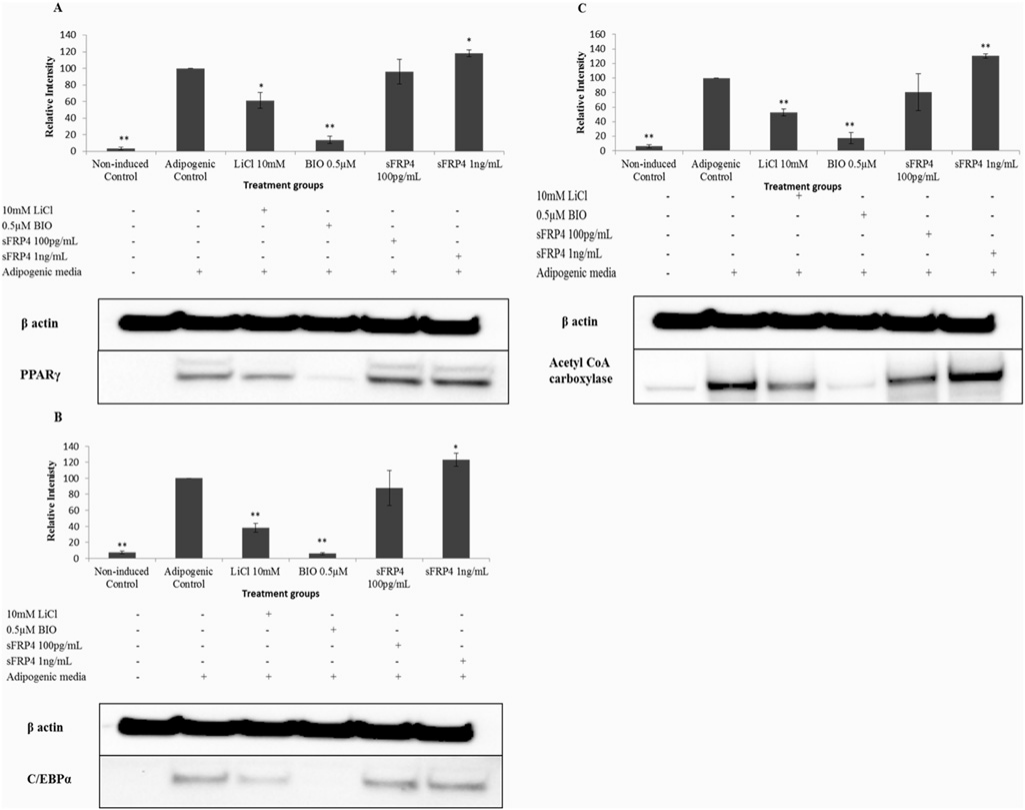
Western blotting results of adipogenesis-specific markers. Representative image of 3 repeats and quantification of (A) PPARγ, (B) C/EBPα, and (C) Acetyl CoA carboxylase adipogenic marker proteins. All values were compared to the adipogenic media control (*p<0.05 and ** p<0.001).

To study the effect of the Wnt regulators on adipogenesis in ADSCs at the protein level, immunoblotting was performed using antibodies against the key adipogenic marker proteins PPARγ, CCAAT enhancer binding protein (C/EBPα), and acetyl CoA carboxylase. sFRP4 at 1ng/mL concentration significantly upregulated the expression of PPARγ by 1.2-fold, C/EBPα by 1.23-fold, and acetyl CoA carboxylase by 1.3-fold respectively (Fig. 5A, 5B, 5C). The expression of PPARγ, C/EBPα, and acetyl CoA carboxylase was decreased significantly with LiCl (by 1.6, 2.6, 1.9-fold respectively) and BIO (by 7, 17, 5.6-fold respectively) treatments (Fig. 5A, 5B, 5C).

The increase in expression of the three key adipogenic marker proteins by sFRP4 (at 1ng/mL) indicated the promoting effect of Wnt antagonism on adipogenesis. On the contrary, the downregulation of adipogenic marker proteins observed in the LiCl- and BIO-treated ADSCs confirmed the inhibitory action of Wnt signalling activation on adipogenesis.

### Effect of combination treatment (B+S) on adipogenic differentiation

**Fig 6:**
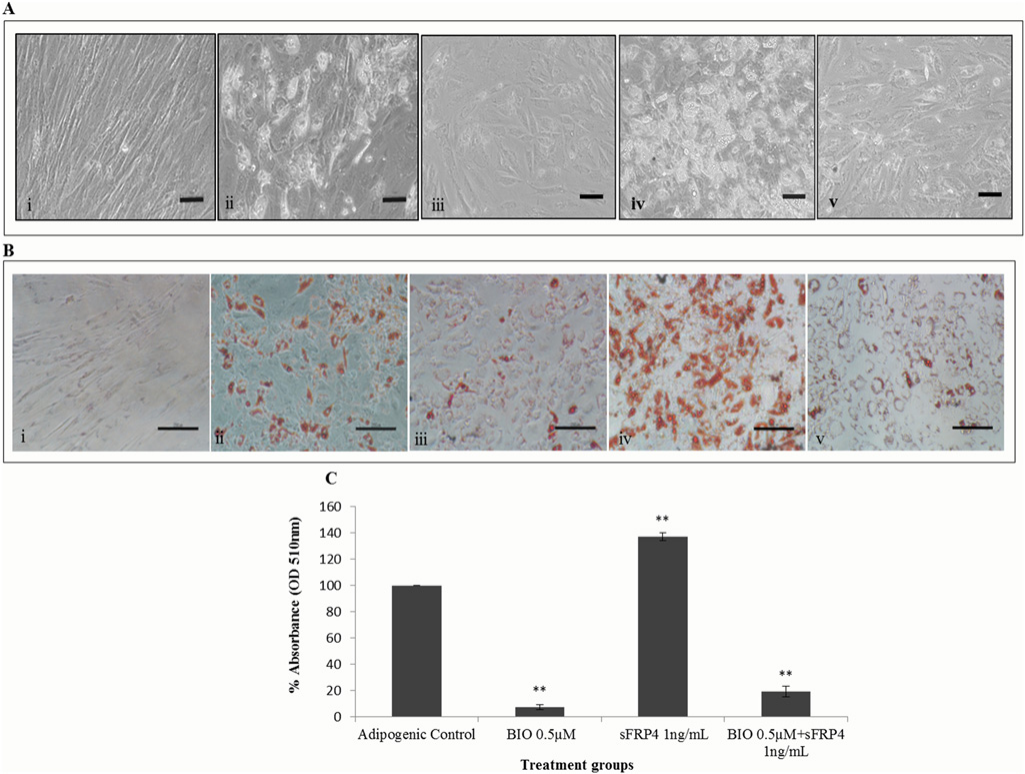
Morphology of ADSCs during adipogenic differentiation at Day 7. (A) Before and (B) after Oil red O staining. Treatment conditions were (i) non-induced control media, (ii) adipogenic control, (iii) 0.5μM BIO, (iv) 1ng/mL sFRP4, and (v) 0.5μM BIO + 1ng/mL sFRP4 in both (A) and (B). Scale bar = 10μM in (A) and 250μM in (B). (C) Quantification of the stained lipid droplets were performed using the eluted Oil red O stain via measuring absorbance at 510nm. The readings were normalised to background values of non-induced control ADSCs. The values of all treatment conditions were compared to the adipogenic control group (** p<0.001).

To study the simultaneous effect of Wnt activator and antagonist on adipogenic differentiation, the degree of lipid accumulation in the presence of both BIO at 0.5μM and sFRP4 at 1ng/mL (B+S) was observed. The morphology of the cells changed from the spindle-like shape to a rounded shape on Day 1 of differentiation in both BIO-only and B+S treatment groups. However, on Day 7 the lipid accumulation remained unchanged in the B+S treatment group when compared to the BIO-only group (Fig. 6A). This was confirmed by Oil red O staining and its quantification (Fig. 6B and 6C), which showed no significant difference in lipid accumulation between BIO-only and B+S treatment groups. This indicated the strong inhibitory action of BIO on adipogenesis which was not reversed by the adipogenic-promoting effect of sFRP4.

## Discussion

Obesity and the metabolic syndrome are major health and economic challenges; so understanding the molecular mechanisms that regulate adipogenic differentiation in humans may give important insight into pathogenesis and treatment of these conditions. Among the several pathways that regulate adipogenesis, the canonical Wnt signalling pathway is thought to be a key player but this complex area has not been fully investigated. In this study we have explored specifically the impact of the Wnt agonists—LiCl and BIO, and the Wnt antagonist sFRP4 in adipogenic differentiation.

The role of Wnt signalling pathway as an adipogenic switch has been established [9] with considerable evidence now indicating the crucial role of Wnt activation in blocking adipogenesis. The inhibitory effect of canonical Wnt ligands such as Wnt-1, and Wnt 10b has been demonstrated on 3T3-L1 preadipocytes [9] and an activated Wnt pathway has been shown to block adipogenic differentiation of pericytes [16]. Few studies have shown the impact of the GSK3β inhibitors on adipogenesis [9,15,17].

Adipose tissue-derived MSC cell lines were used to investigate adipogenesis independently from patient-specific factors. Our cells being adipose tissue-derived possess adipogenesis as their prime physiological purpose [18], and possess higher adipogenic differentiation potential [7,8]. ADSCs could also be the largely recruited population during an adipocyte hyperplasia taking into account their *in vivo* abundance compared to other MSC sources [5,19]. Hence ADSCs were suitable candidates to study the underlying mechanisms of adipogenesis in relation to obesity.

To study the association between the Wnt signalling pathway and adipogenesis, we examined and compared the effect of Wnt activators and a Wnt antagonist on adipogenic differentiation. We chose the Wnt antagonist sFRP4 for the study because it had been found to be highly expressed during adipogenic differentiation [20], making it likely that sFRP4 could be playing a positive role in the adipogenic differentiation. We wanted to investigate this process further, and assess the direct effect of recombinant sFRP4 on adipogenic differentiation of ADSCs. A previous study showed an increase in adiponectin secretion after a short 48-hour treatment with recombinant sFRP4 [21]. Hence, we wanted to see if the continuous supplementation of recombinant sFRP4 throughout the differentiation period would further enhance adipogenesis. Therefore, we carried out sFRP4 treatment for 7 days to detect its effect on lipid droplet accumulation and adipogenic proteins. We also performed continuous supplementation of the Wnt activators LiCl and BIO during adipogenic induction. We showed that after the continuous sFRP4 treatment at 100pg/mL, we observed a decrease in lipid accumulation, which could be attributed to sFRP4 reducing cell viability. However, we found that treatment with 1ng/mL sFRP4 increased lipid droplet accumulation in spite of the effect of sFRP4 on cell viability. We infer from these data that there is a threshold level at which sFRP4’s adipogenic differentiation effects are able to override its effect on reducing cell viability.

We also found that a higher dose of sFRP4 (1ng/mL) was required for upregulating the adipogenesis-specific (PPARγ, C/EBPα, and acetyl CoA carboxylase) protein expressions. PPARγ and C/EBPα are the two key transcription factors responsible for development of a mature adipocyte [22], while acetyl CoA carboxylase is the key enzyme involved in fatty acid biosynthesis. The suppressive effect exerted by the Wnt signalling pathway on these adipogenic regulators [9] was overcome with the sFRP4 treatment in our experiments. We have previously demonstrated a Wnt-inhibitory effect of sFRP4, where sFRP4 treatment (125pg/mL and 250pg/mL) was able to significantly lower the level of nuclear β-catenin in human umbilical vein endothelial cells [23]. In addition, we also demonstrated that sFRP4 abolished the Wnt3a-induced increase in cytosolic β-catenin in mammary epithelial cells [24]. Conversely, we were able to demonstrate that silencing of sFRP4 in A2780 ovarian cancer cells resulted in upregulation of β-catenin expression [25].

In this study, the treatments with LiCl and BIO were performed so that the down-regulating effect of these molecules on adipogenesis could be used as a control against the inducing effect of sFRP4. We demonstrated a significant decrease in the expression of PPARγ, C/EBPα, and acetyl CoA carboxylase proteins in BIO-treated ADSCs. While the effect of LiCl on PPARγ and C/EBPα was evident in previous studies [21], our data demonstrated for the first time its down-regulating effect on the protein levels of acetyl CoA carboxylase in ADSCs. The identical levels of lipid accumulation between the BIO-only treatment and the BIO+sFRP4 combination treatment indicated the strong adipogenic inhibition rendered by activation of Wnt signalling via BIO and also could have been because the effect of BIO is at the intracellular level compared to the extracellular action of sFRP4. Hence, this confirms the necessity of accomplishing Wnt inhibition, which could be carefully considered while engineering adipose grafts for patients undergoing reconstructive surgeries and soft tissue augmentation. Our data provide preliminary insights to the more complex mechanisms underlying adipogenic differentiation and shed light on the key signalling pathway regulating it. However, animal studies and dose optimisation for the *in vivo* conditions will be necessary to further extrapolate the findings.

In summary, this study demonstrated for the first time the promoting effect of recombinant sFRP4 on adipogenic differentiation. And therefore, could be specifically targeted by sFRP4 inhibitors to tightly regulate the development of mature adipocytes from their precursors. This could develop better control over the obesity and related metabolic diseases such as T2 diabetes, wherein sFRP4 levels are shown to be elevated [26]. The findings from our study provide a detailed account of the interplay between the activation and antagonism of the canonical Wnt signalling pathway linked with human adipogenesis. Overall, our study facilitates a better understanding of adipose tissue biology, which is essential to develop new targets to manage diabetes and obesity, together the most preventable and reversible causes of metabolic disorders worldwide.

## Supporting Information

S1 Fig Western blotting results of PPARγ. (A), (C), (E) show images blotted for PPARγ (50Kda), and (B), (D), (F) show images blotted for β-actin (45KDa).
(TIF)

S2 Fig Western blotting results of C/EBPα. (A), (C), (E) show images blotted for C/EBPα (42Kda), and (B), (D), (F) show images blotted for β-actin (45KDa).
(TIF)

S3 Fig Western blotting results of Acetyl CoA Carboxylase. (A), (C), (E) show images blotted for Acetyl CoA Carboxylase (280Kda), and (B), (D), (F) show images blotted for β-actin (45KDa).
(TIF)
