# Supplementary figures and images for: Wnt Antagonist Secreted Frizzled-Related Protein 4 Upregulates Adipogenic Differentiation in Human Adipose Tissue-Derived Mesenchymal Stem Cells
Source: PLoS One. 2015 Feb 25;10(2):e0118005. doi: 10.1371/journal.pone.0118005 (PMC4340908; doi:10.1371/journal.pone.0118005)

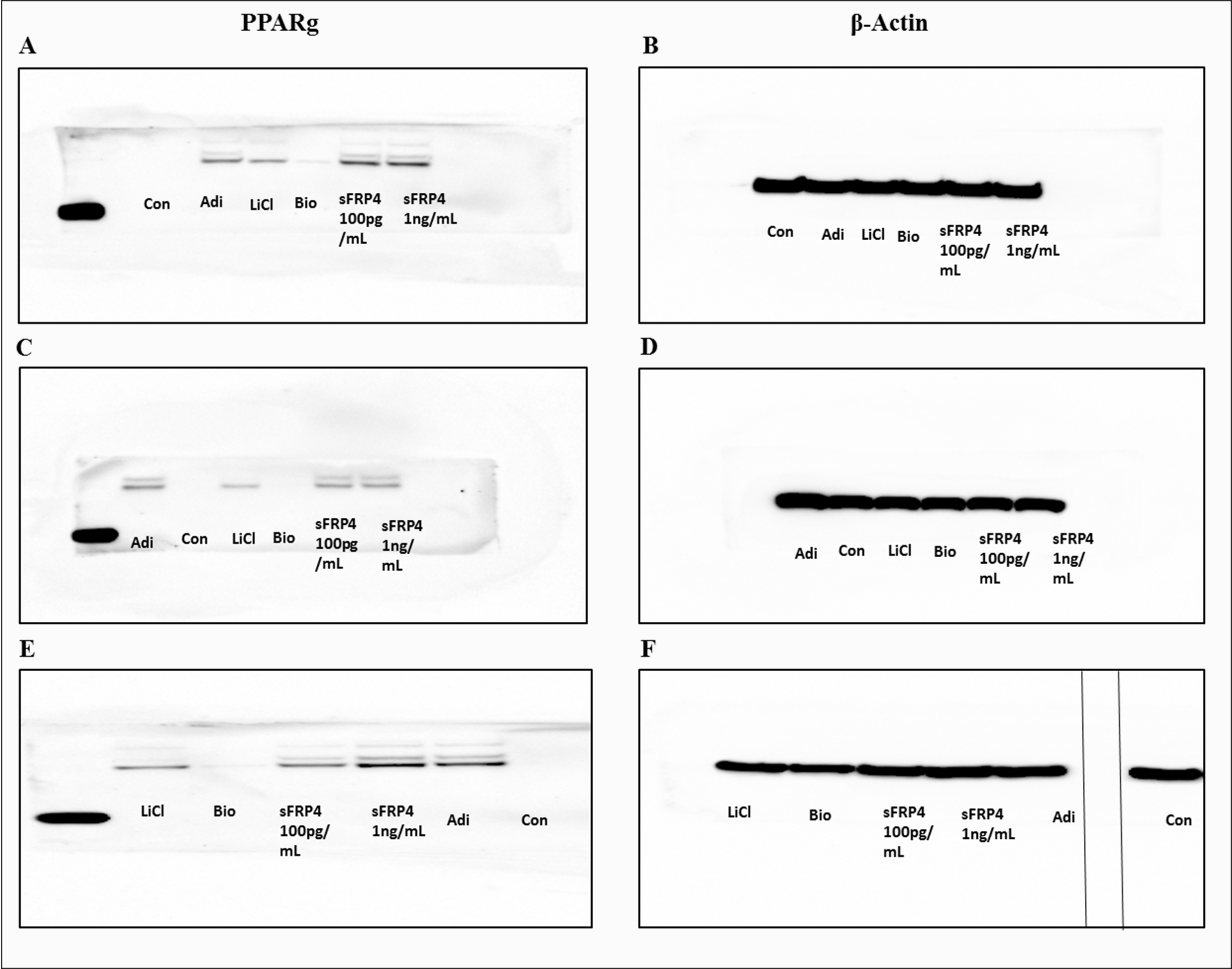

Supplement: S1 Fig — (A), (C), (E) show images blotted for PPARγ (50Kda), and (B), (D), (F) show images blotted for β-actin (45KDa). (TIF) [file pone.0118005.s001.tif]

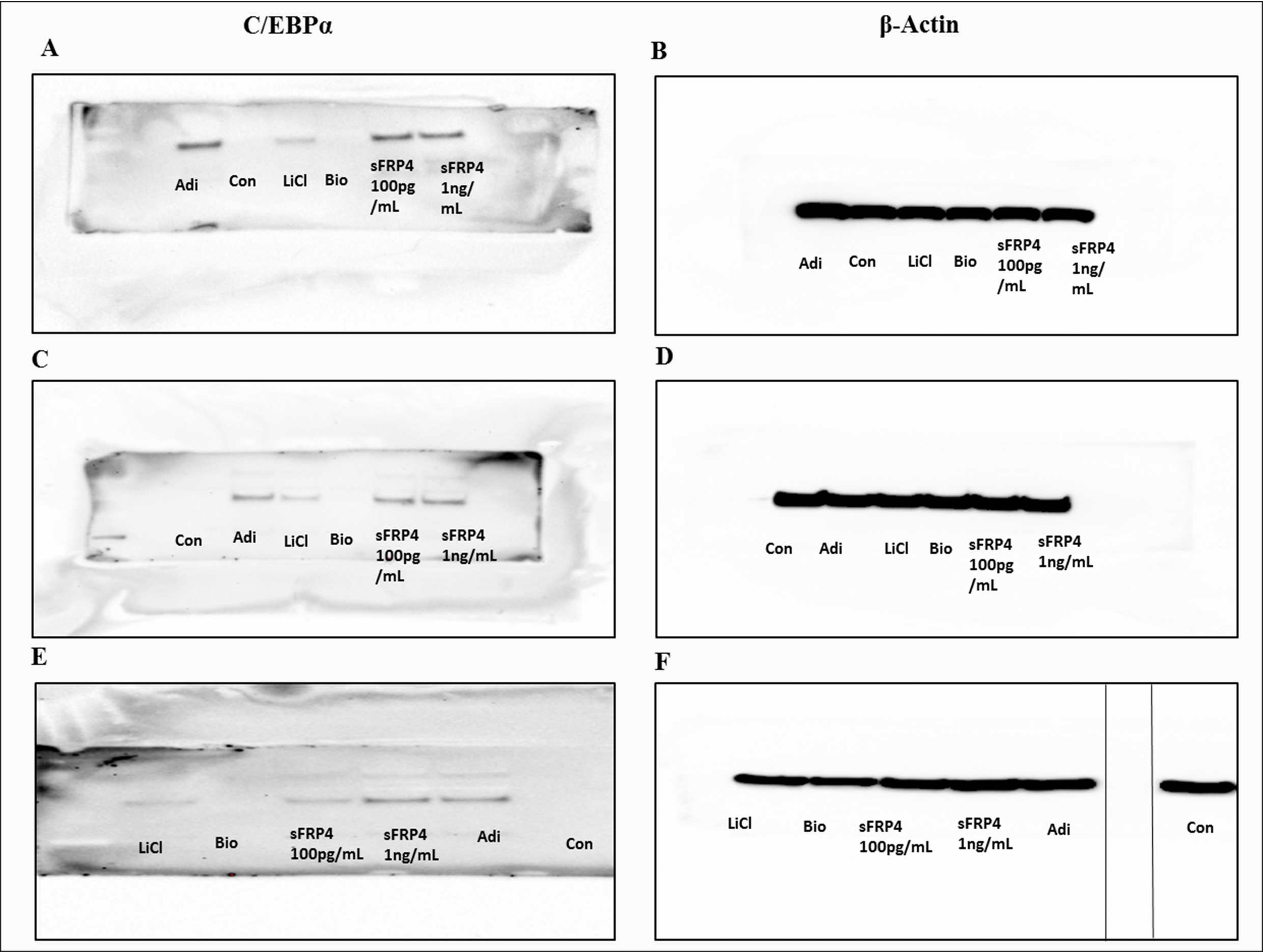

Supplement: S2 Fig — (A), (C), (E) show images blotted for C/EBPα (42Kda), and (B), (D), (F) show images blotted for β-actin (45KDa). (TIF) [file pone.0118005.s002.tif]

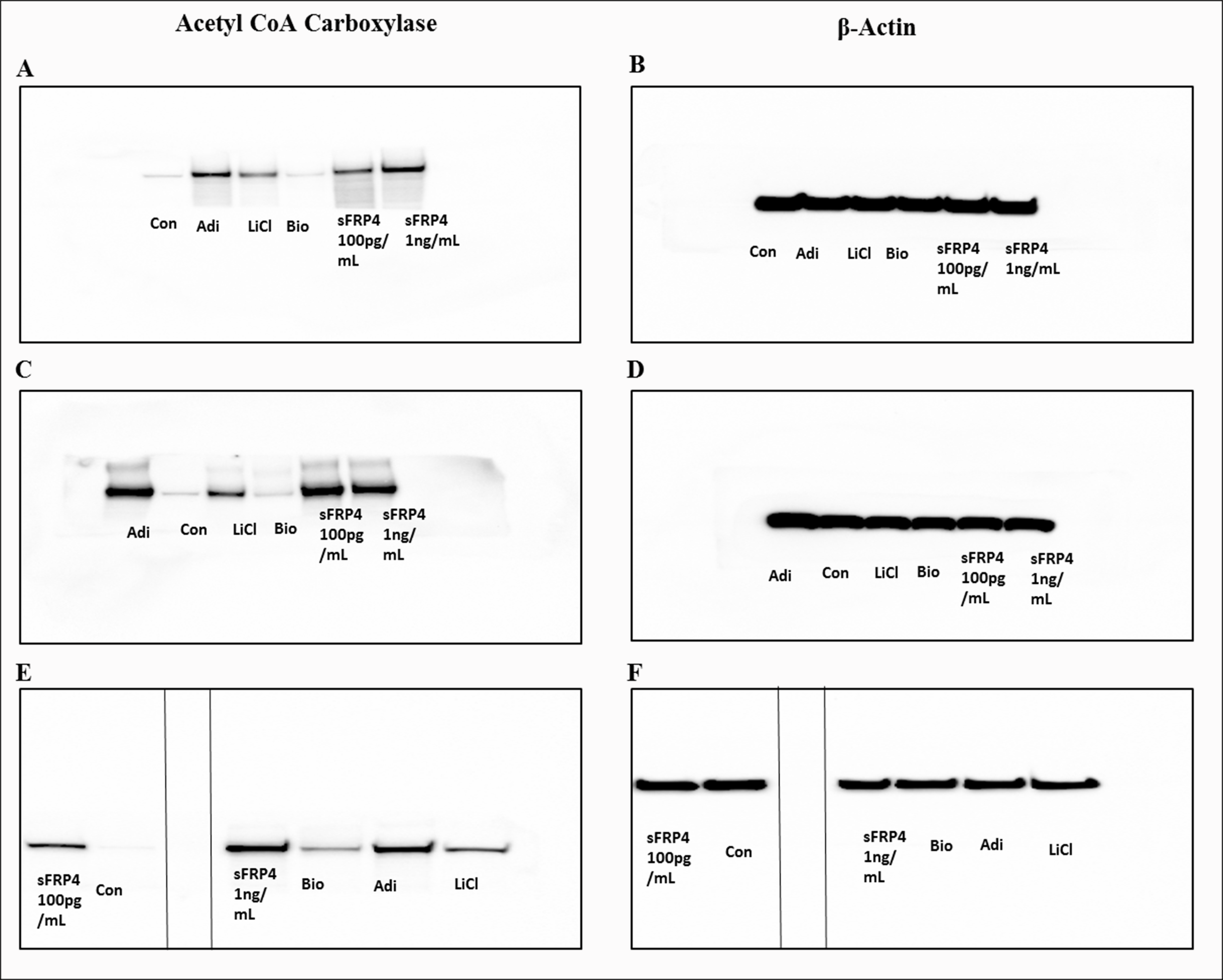

Supplement: S3 Fig — (A), (C), (E) show images blotted for Acetyl CoA Carboxylase (280Kda), and (B), (D), (F) show images blotted for β-actin (45KDa). (TIF) [file pone.0118005.s003.tif]
